# Saccharide mapping apparatus for real-time PAGE detection of polysaccharides

**DOI:** 10.1016/j.jare.2025.03.006

**Published:** 2025-03-04

**Authors:** Baojie Zhu, Jing Zhao, Shaoping Li

**Affiliations:** a State Key Laboratory of Quality Research in Chinese Medicine, Institute of Chinese Medical Sciences, University of Macau, 999078, Macao; b Joint Laboratory of Chinese Herbal Glycoengineering and Testing Technology, University of Macau & National Gly-coengineering Research Center, 999078, Macao; c Macao Centre for Testing of Chinese Medicine, University of Macau, 999078, Macao

**Keywords:** Saccharide-mapping apparatus, Polysaccharides analysis using carbohydrate gel electrophoresis (PACE), Real-time monitor, Quality control, Polysaccharides

## Abstract

**Introduction:**

As a key technique in saccharide mapping, polysaccharides analysis using carbohydrate gel electrophoresis (PACE) analysis platform has always been self-assembled and built in various laboratories independently, but there is no unified commercial analysis instrument platform so far.

**Objective:**

This research aimed to establish an integrated commercially viable PACE analysis platform to standardize the application of PACE in the field of saccharides analysis and the quality control of polysaccharides.

**Methods:**

We established an online detection platform of saccharide-mapping apparatus (SMA) and took advantage of it to characterize the oligosaccharides for quality control of polysaccharides in herbal medicine. The polysaccharides from *Gastrodia elata* Bl. were finally selected to verify the potential of SMA including precision, repeatability, stability, and sensitivity.

**Results:**

The online detection platform of SMA was more integrated and commercially viable, which can investigate oligosaccharides from polysaccharides after hydrolysis more quickly and accurately for quality control. Consequently, it could enable real-time monitoring of oligosaccharide migration, facilitate the determination of the optimal endpoint of polyacrylamide gel electrophoresis (PAGE), and improve the sensitivity of PACE. In addition, the refrigeration device configured in SMA can solve the problem of temperature fluctuation caused by joule heat during gel electrophoresis, which greatly enhances the separation resolution. Finally, the proposed platform was applied to real samples of polysaccharides from *Gastrodia elata* Bl., and the results successfully distinguished the polysaccharides from *Gastrodia elata* Bl. with different cultivars.

**Conclusion:**

The developed online detection platform of SMA offers a rapid and convenient approach for quality control of polysaccharides in Chinese medicines and can be applied for future advancements toward commercialization.

## Highlights

• Commercially viable online detection platforms are crucial for quality control of polysaccharides.
• Developed the Saccharide-Mapping Apparatus (SMA) for real-time detection of polysaccharides.
• SMA is more integrated and commercially feasible to ensure data consistency and comparability.
• Online imaging realizes indicator-free and real-time monitoring of saccharides separation process.
• The SMA has been applied to the quality control of polysaccharides from *Gastrodia elata* Bl.

## Graphical abstract

**Figure:**
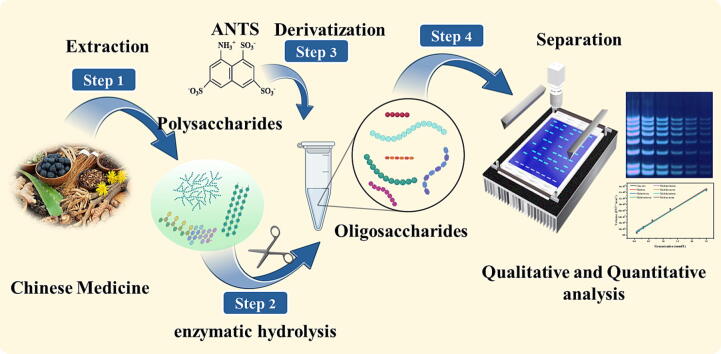

## Introduction

Saccharide mapping is a powerful method for quality control of polysaccharides and has been effectively utilized for qualitative and quantitative analysis of polysaccharides from Chinese medicines [1], [2]. It provides essential technology and demonstration for establishing international standards for Chinese medicines rich in polysaccharides, such as *Lycium barbarum* [3], *Ganoderma lucidum* [4], [5], *Cordyceps sinensis* [6], *Pseudostellaria heterophylla* [7] and etc. As a key technique in saccharide mapping, polysaccharides analysis using carbohydrate gel electrophoresis (PACE) is a sensitive and straightforward method for characterization of polysaccharides, which relies on derivatization of the reducing ends of saccharides with a fluorophore and separation by polyacrylamide gel electrophoresis (PAGE) with optimized conditions [5], [8]. However, PACE analysis platform has always been self-assembled and built in individual laboratories, and there is no unified commercial analysis instrument platform so far [9], [10], [11], [12], [13]. These self-assembled and home-made PACE analysis platforms often struggle to achieve the accuracy and stability of commercial instruments, leading to potential deviations in measurement accuracy, ultimately affecting the reliability of experimental data [12], [14]. Therefore, the establishment of an integrated commercially viable PACE analysis platform is of significant importance for standardizing the application of saccharides mapping in quality control of polysaccharides.

To achieve the goal mentioned above, it is essential to understand and address some of the problems existing in conventional PACE. One notable limitation is the inability to visualize oligosaccharides labeled with fluorescent groups under PAGE analysis due to their colorless property [15]. Therefore, PAGE separation process must rely on migration indicators for monitoring [16] and time-consuming preliminary optimization is often required by comparing the positions of the indicator and sample bands to ensure accurate analysis progress, especially given the complexity of oligosaccharides structures. Furthermore, it is challenging to observe the analysis result of PAGE in real-time, which can lead to the loss of key experimental results during the process, such as the migration process of the sample band during electrophoresis, the diffusion of the band during migration, and the true migration endpoint [5]. Understanding these experimental results allows for the timely adjustment of electrophoresis conditions (such as voltage and current) to optimize sample separation, enhance result clarity, and ensure the bands migrate as expected. This ultimately improves the repeatability and operability of the experiment, making the results more reliable. So, implementing the real-time analysis capability of PAGE is more efficient and accurate in enhancing the overall utility of PACE in saccharide mapping for the analysis of polysaccharides.

## Material and methods

### Reagents and materials

*α*-Amylase, *endo*-Dextranase, Starch (STA) and Dextran (DEX) were obtained from Megazyme (Wicklow, Ireland). D-glucose (Glu), maltose (Mal), maltotriose (Mal-3), maltotetraos (Mal-4), maltopentaose (Mal-5), maltohexaos (Mal-6), maltoheptaose (Mal-7), maltooctaose (Mal-8) were purchased from Aladdin Biochemical Technology Co., Ltd. (Shanghai, China). ANTS (8-Aminonaphthalene-1,3,6-trisulfonic acid disodium salt) was obtained from Tokyo Chemical Industry (Tokyo, Japan). Acrylamide-Bis-Acrylamide Solution (19:1, *w*/*w*) with different concentrations were obtained from Bio-Rad (Hercules, CA, USA). Deionized water was provided from Millipore Milli-Q Plus system (Millipore, Bedford, MA, USA) in all experiments. All the other chemicals and reagents used met analytical-grade standards.

### Preparation of water-soluble polysaccharides

Initially, 80 % ethanol was mixed with the *Gastrodia elata* powder at a ratio 20:1 (*v*/*w*) and incubated at 80 °C for 2 h to remove lipids, colored substances, and small molecules. The extraction solution was further cooled to room temperature and centrifugated at 4500 × *g* for 15 min. The residue was re-suspended in water at a ratio of 20:1 (*w*/*w*) and reflux extracted at 100 °C for 2 h. The extraction solution was finally cooled to room temperature and centrifugated at 4500 × *g* for 15 min. The centrifugate was concentrated under vacuum, precipitated with ethanol at the final concentration of 75 % (*v*/*v*), and centrifuged at 4500 *× g* for 15 min. The resulting precipitate was dissolved in deionized water and filtered through an ultrafiltration membrane (3 kDa cut-off, Millipore, Billerica, MA, USA) to remove low molecular weight compounds. The final polysaccharides with molecular weight greater than 3 kDa were freeze-dried to obtain the water-soluble *Gastrodia elata* polysaccharides (GEPs), which were stored at 4 °C for further analysis.

### Partial acid hydrolysis of polysaccharides

Each GEPs or dextran solution (200 μL, ∼0.4 mg) was mixed with an equal volume of trifluoroacetic acid (TFA, 2 mol·L^−1^) and hydrolyzed at 80 °C for 2 h, following previously described protocols with slight modifications [17]. Finally, the hydrolysates were freeze-dried and derivatized for PACE analysis. GEPs solution was processed as described above but without TFA as a blank control.

### Enzymatic hydrolysis of polysaccharides

For each sample, the GEPs solution (200 μL, approximately 0.4 mg) was depolymerized with selected *α*-amylase and *endo*-dextranase (final concentrations of 20 U·mL^−1^ each) at 40 °C for 12 h. After hydrolysis, the enzymes were inactivated at 80 °C for 10 min, and resulting solids were removed by centrifugation. The hydrolysis products of GEPs were then freeze-dried and derivatized for PACE analysis. Similarly, polysaccharide standards of starch and dextran were treated with *α*-amylase and *endo*-dextranase, respectively. GEPs solutions without enzyme treatment, processed as described, were used as blank controls.

### Derivatization with ANTS

Derivatization was carried out with slight modifications to the reported procedure [15], [18]. ANTS was prepared in acetic acid/water (3:17, *v*/*v*) to a final concentration of 0.1 mol·L^−1^. NaCNBH_3_ was dissolved in DMSO to a final concentration of 1 mol·L^−1^ for the derivatization reaction. Each dried sample was dissolved in 50 μL of ANTS solution and 50 μL of NaCNBH_3_ solution. The reaction mixture was vortexed, centrifuged, and incubated at 37 °C for 17 h. Following incubation, the solution was freeze-dried. The derivatized sugars were re-suspended in an appropriate volume of urea solution (6 mol·L^−1^) and stored at −20 °C until analysis.

### Saccharide-mapping apparatus

**Fig. 1:**
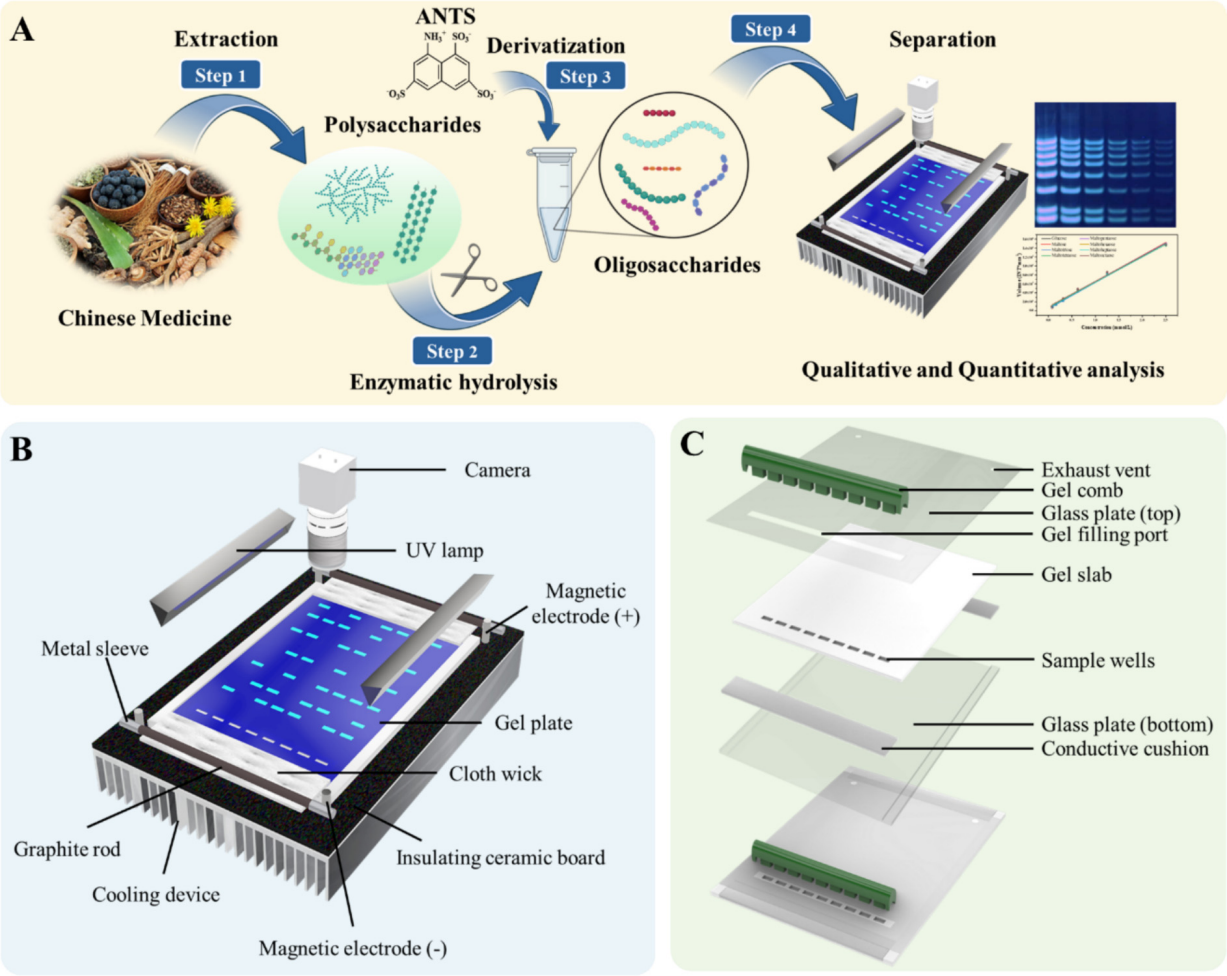
(A) Online detection platform of SMA for samples detection. (B) Schematic illustration of the SMA. (C) Schematic illustration of the planar polyacrylamide gel plate.

Saccharide mapping electrophoresis apparatus (SMA) was designed and developed based on the semi-dry planar polyacrylamide gel electrophoresis by our group in the laboratory, which can realize real-time and quantitative analysis of oligosaccharides. The image system of SMA consisted of an LED light source (365 nm), a charge-coupled device (CCD) camera with a resolution of 5 million pixels and 365 nm filters. Thanks to the advantages of CCD cameras such as high photoelectric conversion efficiency (Quantum Efficiency, QE), low noise, high dynamic range and lossless transmission, it can capture more details and provide richer image information in low light environments and long exposures, thus ensuring high image quality. The SMA applies voltage for electrophoresis analysis to the gel by contacting the graphite rod with the cloth wick soaked in electrophoresis fluid. The magnetic electrode column installed on the SMA can firmly adsorb and fix the graphite rod with metal blocks at both ends through magnetic force, ensuring stable contact between the graphite rod and the cloth core, while facilitating disassembly. As well as that, SMA is equipped with a traditional and cheap water-cooled temperature control device. The cold head (water block) of the cooling system is positioned beneath the ‘insulating ceramic board’ of the SMA, ensuring a tight fit (Fig. 1B). During operation, the temperature is adjusted according to experimental requirements, and the ‘gel plate’ placed on the ‘insulating ceramic board’ helps regulate the Joule heat generated during electrophoresis through continuous heat exchange. Finally, all samples (1.0–3.0 μL depending on the sample concentration) were separated using SMA with the final optimized condition at 10 °C in all cases. In addition, various conditions were tested during the electrophoresis analysis to achieve the optimum separation condition, including the percentage of acrylamide in gels, electrophoresis buffer concentrations, and voltages.

### Preparation of planar polyacrylamide gels

The planar polyacrylamide gel separation is performed using SMA equipment with a new mold for pouring gel (Fig. 1C). Prior to assembling the gel casting system, the gel cassette was meticulously cleaned with a lint-free tissue and detergent, followed by thorough rinsing with ultrapure water. The resolving gel consists of polyacrylamide with 0.04 % (*v*/*v*) N,N,N′,N′-tetramethylethylenediamine (TEMED) and 10 % (*w*/*v*) ammonium persulphate in Tris-borate buffer. After thoroughly mixing the acrylamide solution, it was poured into the gel filling port of the glass plates (top), and a gel comb was positioned before polymerization commenced. The conductive cushion mentioned in Fig. 1C can fit tightly around the gel pouring mold to ensure that there will be no leak during the gel pouring process. At same time, the exhaust vent on the upper glass plate of the gel casting mold can ensure that all bubbles in the casting process will eventually be discharged through this hole. Finally, 1.5 mm thick planner polyacrylamide gels were successfully prepared for saccharide mapping analysis by SMA.

### Calibration curve

A mixed reference standards stock containing D-glucose, maltose, maltotriose, maltotetraos, maltopentaose, maltohexaos, maltoheptaose, and maltooctaose which have been derivatized by ANTS was prepared by dissolving in urea (6 mol·L^−1^) solution with each concentration at 2.5 mmol·L^−1^. An appropriate volume of the reference stock solution was aliquoted using a pipette and sequentially diluted with urea (6 mol·L^−1^) solution to obtain mixed reference solutions of varying concentrations, which were then measured using SME. Then, a linear regression equation was established using the peak volume (average OD of the band times band area, int*mm^2^ [19]) and the concentration of the analyte in the standard solution (expressed in mmol·L^−1^).

### Method validation

The instrument precision was assessed by generating 6 consecutive bands from the same sample on a gel plate and analyzing the sample across 3 distinct gel plates. Repeatability was evaluated using three aliquots of the selected samples prepared by different hydrolysis methods, analyzed simultaneously. To evaluate sample stability, hydrolysis products of selected samples were stored at −20 °C post-partial acidic hydrolysis and subjected to electrophoresis on the 1st, 3rd, and 5th days. Finally, stock solutions of reference compounds (Glu, Mal, Mal-3, Mal-4, Mal-5, Mal-6, Mal-7, Mal-8) were diluted with a urea solution (6 mol·L^−1^) to various concentrations to determine the limit of detection (LOD) and limit of quantification (LOQ) based on the visual bands of the mixed standards at the lowest concentration.

### Data analysis

Electronic images of PACE fingerprints and optical density of bands in digital scanned chromatograms were generated and analyzed using Quantity One software (version 4.6.2, Bio-Rad, Hercules, USA). Similarity and simulated average chromatograms of test samples were calculated and generated using specialized software called “Chinese Medicine Chromatographic Fingerprint Similarity Evaluation System” (Matlab version, developed by the Modernization of Chinese Medicine Research Center at Central South University and The Hong Kong Polytechnic University, version 315). All statistical analyses were conducted using SPSS statistics software version 26.0 (IBM, Armonk, New York).

## Results and discussion

### Construction of SMA for sample analysis

A rapid and accurate online detection platform is crucial for quality control of polysaccharides in Chinese medicines by saccharide mapping based on PACE. However, significant challenges exist in establishing an online detection platform establishment of the original PACE analysis platform, which relies on traditional wet vertical electrophoresis. The original PACE analysis platform does not allow for real-time monitoring, relying only on indicators to track the analysis process, and gel images are always captured offline after electrophoresis is complete. To improve the online detection of PACE-based saccharide mapping, PACE-based SMA platform was developed, which can realize real-time monitoring and high-throughput quantitative analysis of oligosaccharides in saccharide mapping (Fig. 1A and Fig. S1). In the new SMA platform, the traditional wet gel vertical electrophoresis is replaced by the semi-dry planar gel electrophoresis, featuring an online imaging system and cooling unit (Fig. 1B). The semi-dry planar gel electrophoresis uses a UV light source (365 nm), which can directly illuminate the planar gel through the front glass plate, without interference from the tank walls or buffer solution. Additionally, a cooling system installed below the gel plate effectively absorbs the Joule heat generated during gel electrophoresis analysis, maintaining the gel at a constant temperature. More importantly, compared to the original PACE method with the traditional wet vertical gel electrophoresis, the SMA significantly reduces the amount of electrophoresis solution required, needing only a small amount of solution (about 5 mL) to keep the gel wet for completing each electrophoresis analysis.

### Development and optimization of electrophoresis

**Fig. 2:**
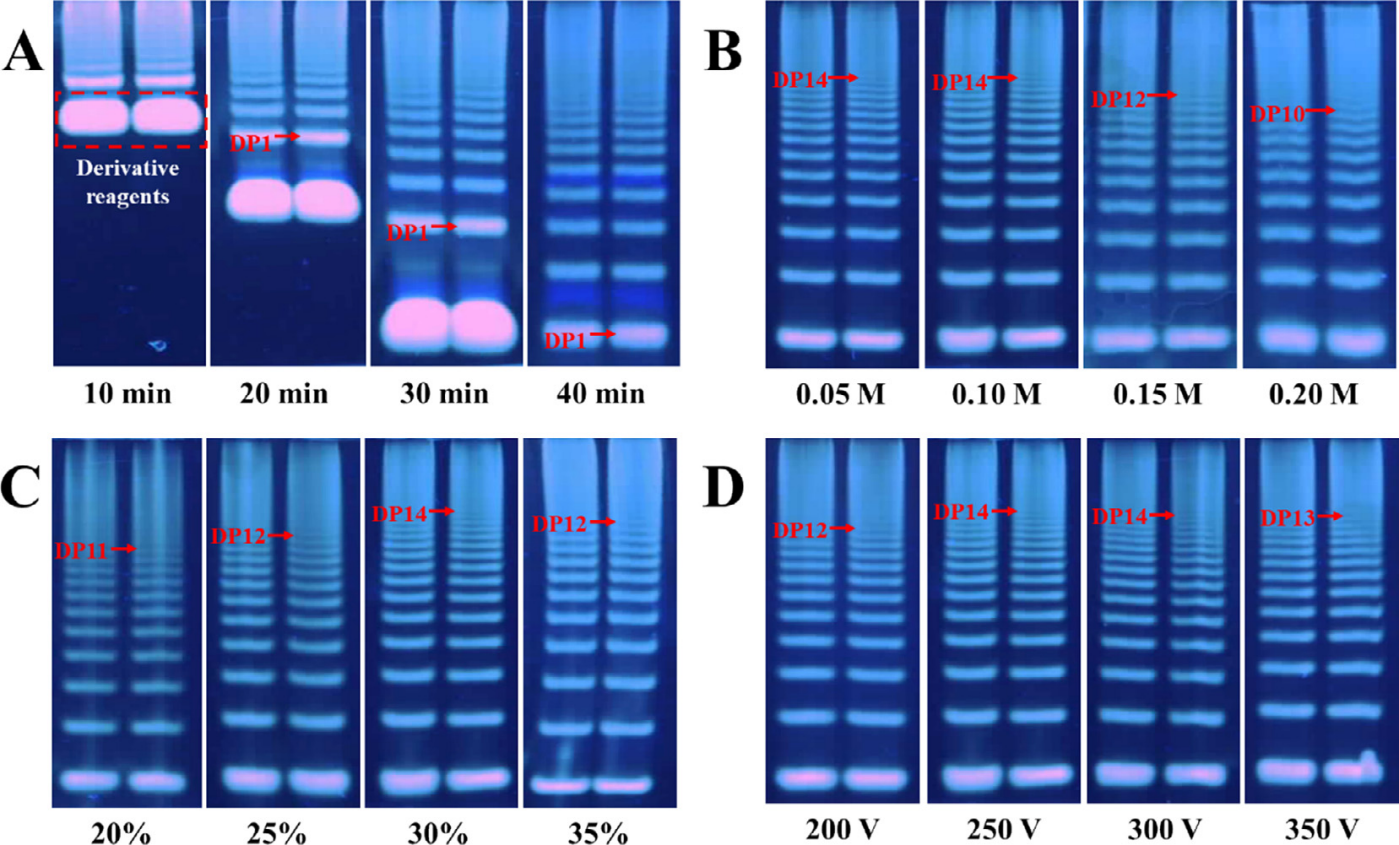
(A) Real-time detection of oligosaccharides migration during the 40 min PACE run by SMA. (B-D) PACE analysis of the partial acid hydrolysates of dextran with concentration of electrophoresis buffer, percentage of polyacrylamide and different voltages.

The online detection platform of SMA is utilized to characterize the oligosaccharides, providing a basis for quality control of polysaccharides in herbal medicines. After casting gel, loading the prepared samples, and loading the running buffer, the SMA enables the simultaneous PACE operation and real-time monitoring of the planar polyacrylamide gel (Fig. 1B). The well-designed SMA allows real-time observation of the oligosaccharides migrating from the sample wells to the gel bottom (Fig. 2 A). This capability enables direct visualization of the separation process, allowing precise determination of the separation endpoint and mitigating the effects of oligosaccharide band diffusion on sensitivity. In contrast, the conventional PACE method does not provide visual access to the separation process, requiring the use of specific indicators to ascertain the endpoint of separation [5]. For achieving the optimum separation condition, the hydrolysates of dextran after partial acid hydrolysis were selected for optimizing the percentage of acrylamide in gels, electrophoresis buffer concentrations, and voltages according to our previous study [5]. As showed in Fig. 2B, the concentration of electrophoresis buffer (Tris–boric acid) plays a critical role in the effective separation of oligosaccharides. A concentration that is too high hinders the separation, while a concentration that is too low results in prolonged electrophoresis time. Similarly, the percentage of polyacrylamide was crucial for effective separation of oligosaccharides, with higher percentage of polyacrylamide can improve the resolution (increasing number of bands from 11 to 14). However, excessively high percentage of polyacrylamide (35 %) complicates gel preparation and preservation, and greatly extends the analysis time, which will ultimately reduce the resolution (decreasing number of bands from 14 to 12) (Fig. 2C). Finally, the increasing voltage would also decrease running time and enhance the separation, which could detect 14 bands at 250 V and 300 V. However, high voltage (350 V) induced decreased resolution because of Joule heating (Fig. 2D). Therefore, the electrophoresis buffer (Tris–borate) with 0.1 mol·L^−1^, percentage of polyacrylamide with 30 % and voltage of 250 V was selected as the final condition for analysis of oligosaccharides with a run time of about 40 min.

### Development and validation of SMA

**Fig. 3:**
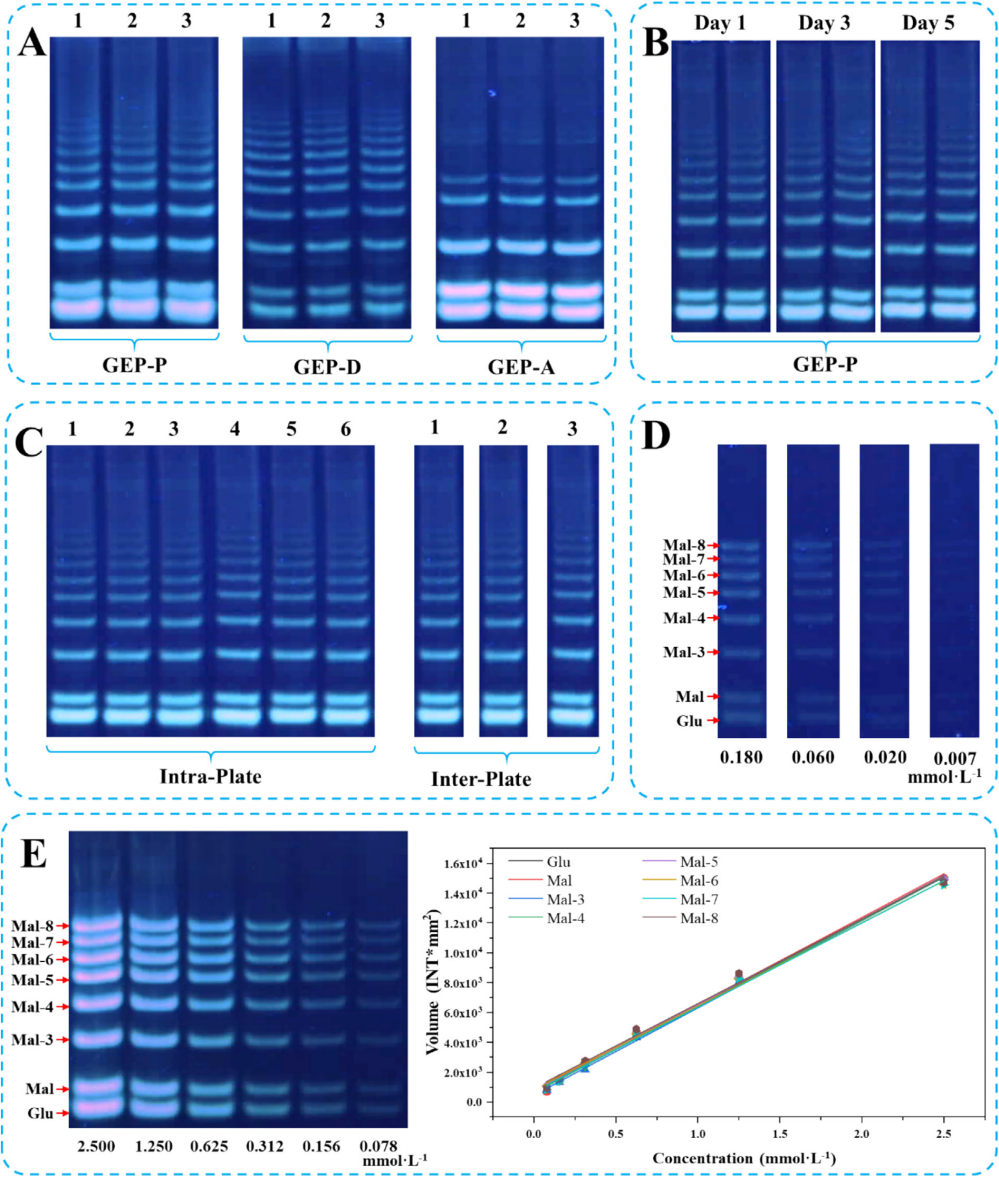
The method validation of repeatability (A), stability (B), precision (C) and limitation of detection and quantification (D), and calibration curve (E). **GEP-P:** hydrolysates from GEPs by partial acid hydrolysis with TFA, **GEP-D:** hydrolysates from GEP by hydrolysis with *endo*-dextranase, **GEP-A:** hydrolysates from GEP by hydrolysis with *α*-amylase.

To verify the potential of SMA, the polysaccharides from *Gastrodia elata* Bl. was selected for detection. It is well-known that precision, repeatability, stability, and sensitivity are critical for developing analytical methods. As shown in Fig. 3A, C, the results demonstrate that the SMA exhibits good precision and reproducibility, with standardized difference values among different oligosaccharide fingerprints being less than 5 %. Additionally, the standard deviation of the oligosaccharide fingerprint of the same sample stored at −20 ℃ for 1, 3, and 5 days was < 5 %, indicating that the sample remains stable for up to 5 days (Fig. 3B). Actually, samples intended for PACE analysis can be stably stored at −20 °C for at least 6 months [17]. The excellent reproducibility and sensitivity provide a robust theoretical foundation for the development of SMA as a quantitative analytical tool. Using Glu and malt oligosaccharides as the model target, the results of LOD at 0.02 mmol·L^−1^ and LOQ at 0.06 mmol·L^−1^ for Glu, Mal, Mal-3, Mal-4, Mal-5, Mal-6, Mal-7 and Mal-8 showed the method has a good sensitivity which higher than the ELSD and RID (Fig. 3D). Accordingly, we also established the linear relationship between the Glu, Mal, Mal-3, Mal-4, Mal-5, Mal-6, Mal-7 and Mal-8 concentration and gray scale (Fig. 3E and Table S1) with coefficient of determination (R^2^) more than 0.997. In addition, given the challenges associated with obtaining purified reference oligosaccharides, a quantification strategy using quantification of multiple components by one marker (QMCOM) or single standard to determine multi-components (SSDMC) was employed to quantify specific oligosaccharides [20], [21]. We also found an interesting manifestation that the curves of selected oligosaccharides with different degrees of polymerization almost overlap. This also means that for a series of similar oligosaccharides with different degrees of polymerization, a “one test multiple evaluation “method can be used for quantitative analysis, which will solve the problem of difficulty in obtaining oligosaccharide reference materials (Table S1).

*Digested polysaccharides fingerprints of GEPs after partial acid and enzymatic hydrolysis using SMA*.

After we validated the optical performance of the SMA, the real-time monitoring of the separation process was firstly demonstrated for oligosaccharides from GEPs after partial acid hydrolysis with TFA and enzymatic hydrolysis with *endo*-dextranase, and *α*-amylase. This device is capable of simultaneously processing 10–20 samples within 40 min (depending on the gel width and the number of sample wells on gel). The partial acid hydrolysates of GEPs from different cultivars including Wuhongtianma (WHTM), Hongtianma (HOTM), and Wutianma (WUTM), which did not exist in the samples before hydrolysis (Fig. S2A), were greatly similar in PACE (Fig. S2B) fingerprints. Furthermore, the similarities of each sample to their simulative chromatogram were evaluated based on the PACE fingerprints (Fig. S2B). The average correlation coefficient between the chromatograms of partial acid hydrolysates of GEPs to their simulated average chromatogram (SMC-H) was 0.986 ± 0.006 (*n* = 22, Table S1), indicating a high similarity in the PACE fingerprints of the acid hydrolysis products from the GEPs (with an average correlation coefficient above 0.90). However, partial acid hydrolysis is non-specific and reflects the overall structural information of the polysaccharides. Therefore, further investigation into enzyme hydrolysis based on polysaccharide chemical structural features was conducted.

Previous studies have shown that GEPs usually consist of glucose with the major glycosidic linkages of *α*-1,4-Glc*p* and *α*-1,6-Glc*p* [22]. Therefore, *endo*-dextranase and *α*-amylase were further selected for digesting GEPs from different cultivars. The results revealed that GEPs in all samples were successfully digested to produce small sugars by the selected enzymes (Fig. S1C, D), and no small sugar molecules were present in the samples before enzymatic treatment (Fig. S1A). The positive responses of endo-dextranase and α-amylase on GEPs suggested that α-1,4-glucosidic and α-1,6-glucosidic linkages existed in all samples, respectively. The average correlation coefficients of each chromatogram of endo-dextranase and α-amylase digested GEPs to their simulative mean chromatograms (SMC-D and SMC-A) were 0.950 ± 0.018 (*n* = 22), and 0.993 ± 0.005 (*n* = 22), respectively, which confirmed that enzymatic fingerprints of GEPs from different cultivars were similar (Table S1).

### Semi-quantitative analysis of the saccharide mapping based on selected oligosaccharide markers

**Fig. 4:**
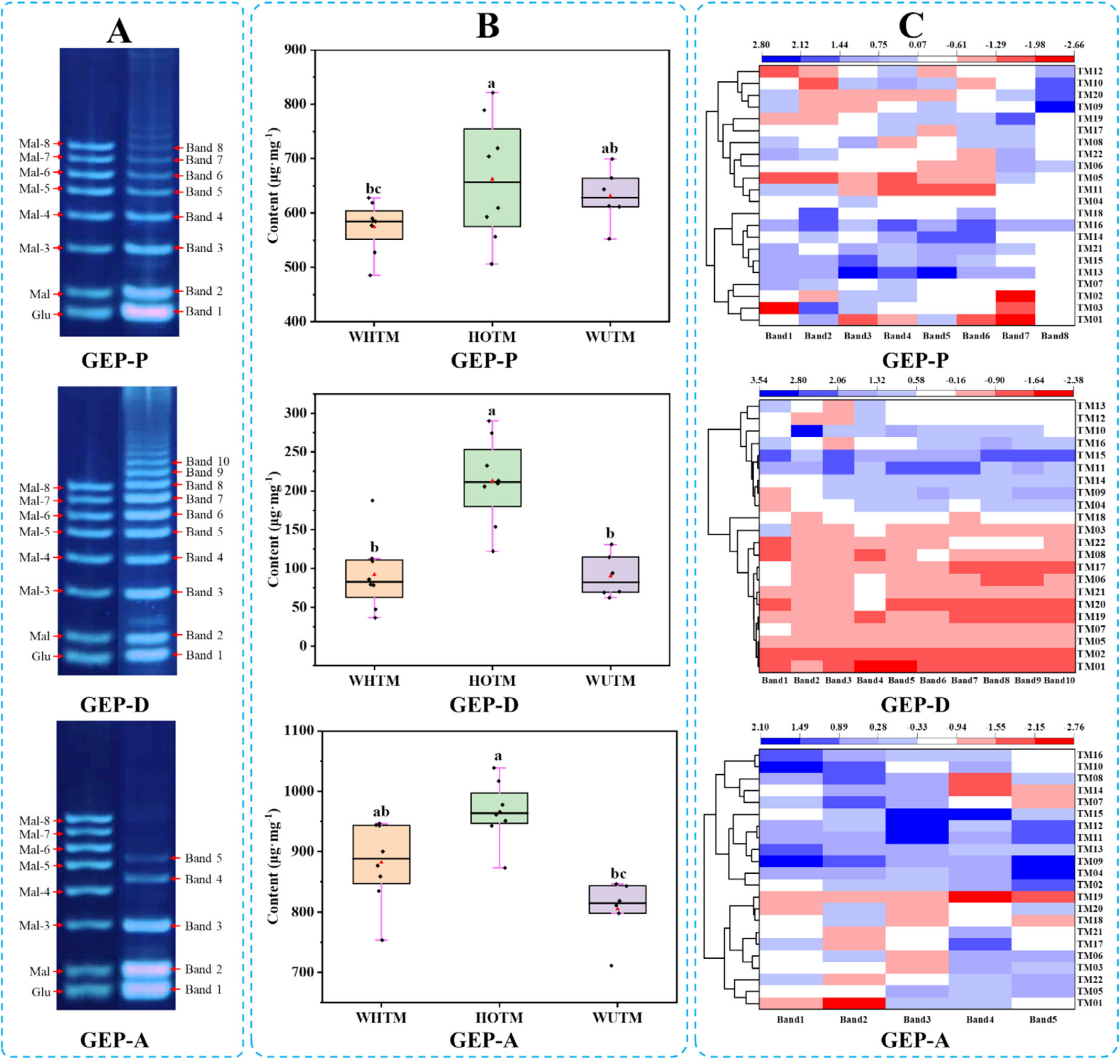
(A) Electropherograms of oligosaccharides standards and oligosaccharides from GEPs after hydrolysis based on SMA. (B) Total content of oligosaccharides from GEPs of different cultivars after hydrolysis. (C) HCA heatmap visualized the relationship between *Gastrodia elata* Bl. samples of different cultivars and oligosaccharide markers. **GEP-P:** hydrolysates from GEPs by partial acid hydrolysis with TFA, **GEP-D:** hydrolysates from GEP by hydrolysis with *endo*-dextranase, **GEP-A:** hydrolysates from GEP by hydrolysis with *α*-amylase. Different letters indicated significant differences between *Gastrodia elata* Bl. samples of different cultivars (*p* < 0.05).

Comparing the positions of oligosaccharides from GEPs after hydrolysis with those of glucose and malto-oligosaccharides with different degrees of polymerization (DP), the bands of oligosaccharides with different DP in hydrolysates from GEPs after partial acid hydrolysis (DP1-DP8), enzymatic hydrolysis with *endo*-dextranase (DP1-DP10) and *α*-amylase (DP1-DP5) were chosen for semi-quantitative analysis (Fig. 4A). To further confirm the structure of oligosaccharides from GEPs after hydrolysis, we further treated them with *β*-amylase and *γ*-amylase and then compared the results with PACE (Fig. S3). The results showed that the oligosaccharides bands from GEPs after partial acid hydrolysis and *endo*-glucosidase hydrolysis consisted of glucose and a series of malto-oligosaccharides, while the first three bands (from bottom to up) of GEPs hydrolysis products after *α*-amylase treatment correspond to glucose, maltose, and maltotriose. The last two bands (from up to bottom) are unidentified oligosaccharides.

To further investigate the difference of GEPs from different cultivars, semi-quantitative analysis of different oligosaccharide markers was conducted using the QMCOM strategy based on the standard curve of glucose and the relative correction factors of the selected oligosaccharides (Table S2–S4). Specifically, the tagged-image format file (TIFF)-based images of different hydrolysates from GEPs after mild hydrolysis by TFA and selected enzymes were analyzed using Quantity One gel analysis software for obtaining the peak volume (int*mm2) of oligosaccharides in individual bands. Then the final oligosaccharide concentration in the individual bands was calculated with the peak volume (int*mm2) substituted into the glucose standard curve and multiplied by the corresponding correction factor and dilution factor (Table S2–S4). It could be seen from Table S2–S4 that the contents of oligosaccharides of GEPs from different cultivars after hydrolysis with different hydrolysis methods had some differences, especially hydrolysis with *endo*-dextranase. Based on analysis of variance done for the total contents of oligosaccharides from GEPs after hydrolysis by *endo*-dextranase, the GEPs from HOTM were also significantly higher than WUTM and WHTM. However, the total contents of oligosaccharides from GEPs from WUTM and WHTM after partial acid hydrolysis and enzymatic hydrolysis showed no significant difference (Fig. 4B).

In order to visually display the relationship between *Gastrodia elata* Bl. samples and selected oligosaccharide markers from GEPs of different cultivars after partial acid hydrolysis and enzymatic hydrolysis, hierarchical cluster analysis was performed based on content of released oligosaccharides from GEPs of different cultivars with different hydrolysis methods (Fig. 4C). The results showed that polysaccharides from WHTM, HOTM and WUTM cannot be well distinguished according to the released oligosaccharides after hydrolysis with TFA. However, it can distinguish HOTM from WHTM and WUTM according to the content of released oligosaccharides from GEPs after hydrolysis by *endo*-dextranase and *α*-amylase, especially hydrolysis by *endo*-dextranase. In addition, the hybrid variety of WHTM is classified under the same category as WUTM in classification, indicating that polysaccharides from WHTM are more similar to WUTM.

## Conclusion

Overall, we integrated existing technologies and developed an online detection platform of SMA that can realize real-time detection of oligosaccharides from polysaccharides after hydrolysis by selected *endo*-enzymes for quality control. Then, polysaccharides from *Gastrodia elata* Bl. was chosen as the model analyte and further method validation verified the feasibility of qualification and quantification of polysaccharides from Chinese medicines. As an integrated brand-new device, the present fully integrated online platform exhibits a series of outstanding characteristics: (1) Compared with the previously self-built PACE analysis platform, SMA is more integrated, commercialized, and more standardized in operation, ensuring higher consistency, comparability, and accuracy of data [12], [14]. At the same time, SMA can perform analysis at a significantly lower voltage (250 V), greatly reducing the impact on the sample, and its analysis time is shortened to only 40 min [3], [5]. (2) Solved the problem of original PACE being unable to observe the analysis process in real time, developed SMA can observe the analysis process of samples in real time. (3) In SMA, traditional wet vertical electrophoresis was replaced with semi-dry planar electrophoresis makes the gel plate better contact with the cooling device to achieve heat exchange, which can accurately control the analysis temperature and avoid the strip dispersion problem caused by Joule heat in the process of gel electrophoresis analysis. These highlights the adaptability of the platform for a broad spectrum of applications in quality control of polysaccharides and oligosaccharides. Combining all these unique and attractive features renders the online platform of SMA very resilient, promising, and captivating for applications in quality control of polysaccharides from Chinese medicines and glycan monitoring.

## Author contributions

**Baojie Zhu:** Investigation, Methodology, Formal analysis, Writing-Original Draft. **Jing Zhao:** Conceptualization, Resources, Writing − Review & Revision, Supervision, Project administration, Funding acquisition. **Shaoping Li:** Conceptualization, Resources, Writing − Review & Revision, Supervision, Project administration, Funding acquisition.

## Compliance with ethics requirement

This article does not contain any studies involving human or animal subjects. Therefore, no ethical approval or informed consent is required. The research adheres to all applicable ethical standards.

## Declaration of competing interest

The authors declare that they have no known competing financial interests or personal relationships that could have appeared to influence the work reported in this paper.
