# Supplementary material for: Saccharide mapping apparatus for real-time PAGE detection of polysaccharides
Source: J Adv Res. 2025 Mar 4;79:151–9. doi: 10.1016/j.jare.2025.03.006 (PMC12766247; doi:10.1016/j.jare.2025.03.006)
Supplement: Supplementary Data 1 [file mmc1.pdf]

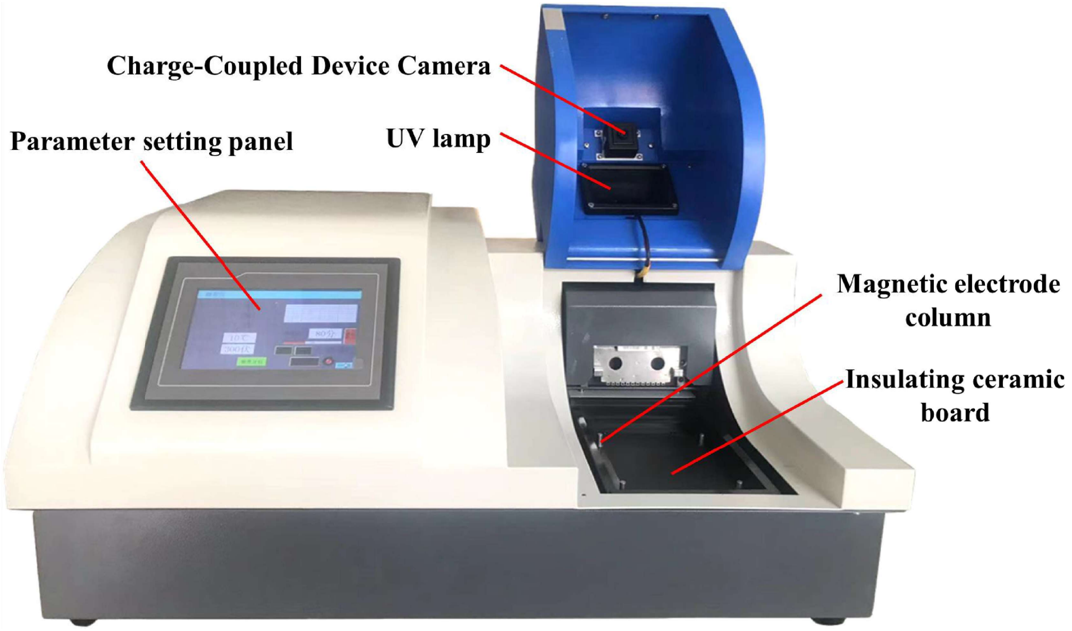

Fig. S1 Physical picture of the first version of “Saccharide Mapping Apparatus”

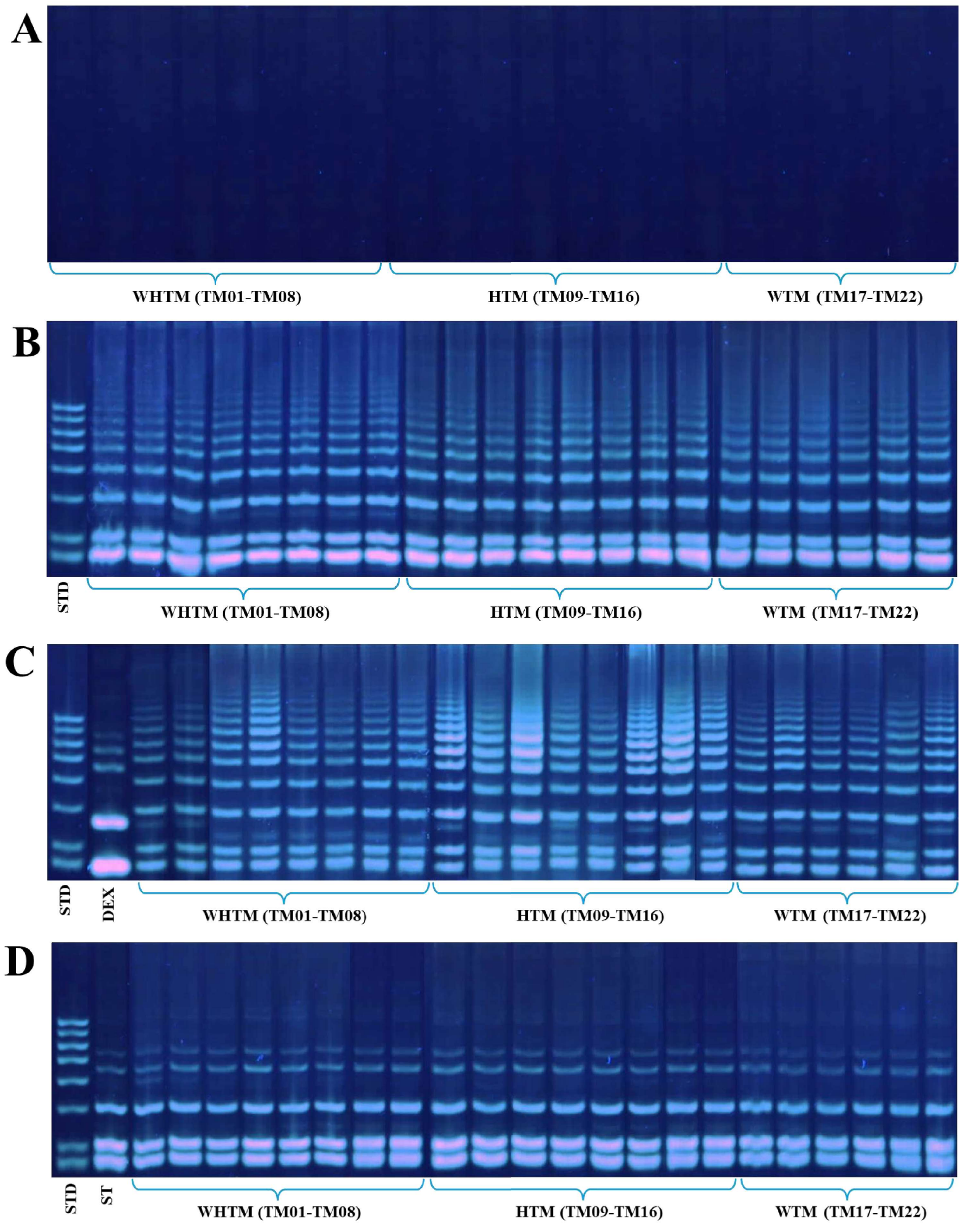

Figure S2 PACE fingerprints of polysaccharides **(A)** and partial acid hydrolysates **(B)** *endo*-dextranase hydrolysates **(C)** and α-amylase hydrolysates **(D)** from polysaccharides in *Gastrodia elata* Bl. **STD** (from down to up), Glu, Mal, Mal-3, Mal-4, Mal-5, Mal-6, Mal-7 and Mal-8; **DEX**, enzymatic digestions of dextran used as marker; **ST**, enzymatic digestions of starch used as marker.

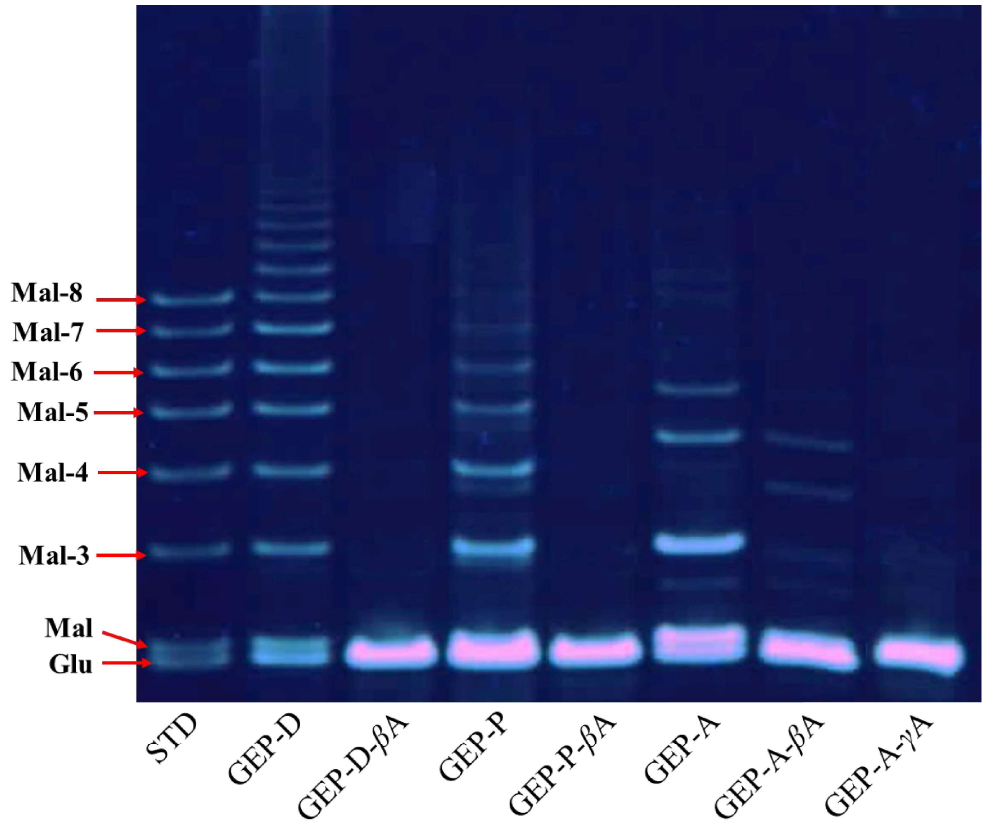

Figure S3 PACE fingerprints of oligosaccharides from polysaccharides in *Gastrodia elata* Bl after hydrolysis with trifluoroacetic acid and different enzymes. **STD** (from down to up), Glu, Mal, Mal-3, Mal-4, Mal-5, Mal-6, Mal-7 and Mal-8; **GEP-P**: hydrolysates from GEPs by partial acid hydrolysis with TFA, **GEP-P-*β*A**: hydrolysates from GEPs by partial acid hydrolysis with TFA and β-amylase, **GEP-D**: hydrolysates from GEPs by hydrolysis with endo-dextranase, **GEP-D-*β*A**: hydrolysates from GEPs by hydrolysis with endo-dextranase and β-amylase, **GEP-A**: hydrolysates from GEPs by hydrolysis with α-amylase, **GEP-A-*β*A**: hydrolysates from GEPs by hydrolysis with α-amylase and β-amylase, **GEP-A-*γ*A**: hydrolysates from GEPs by hydrolysis with α-amylase and γ- amylase.
